# Incidence of mandibular fractures in black sea region of Turkey

**DOI:** 10.4317/jced.52169

**Published:** 2015-07-01

**Authors:** Cihan Bereket, İsmail Şener, Erman Şenel, Nilüfer Özkan, Nergiz Yilmaz

**Affiliations:** 1 DDS, PhD, Associate Professor, Department of Oral and Maxillofacial Surgery, Faculty of Dentistry, Ondokuz Mayıs University, Samsun- Turkey; 2 DDS, PhD, Research Assistant, Department of Oral and Maxillofacial Surgery, Faculty of Dentistry, Ondokuz Mayıs University, Samsun- Turkey; 3 DDS, PhD, Professor, Department of Oral and Maxillofacial Surgery, Faculty of Dentistry, Ondokuz Mayıs University, Samsun- Turkey

## Abstract

**Background:**

The aim of this study is to review the incidence of mandibular fractures in the Black Sea Region of Turkey and to present our treatment protocol.

**Material and Methods:**

Data were collected regarding age, sex, etiology, time distribution, site of the fracture and the associated injuries and evaluated. These patients were treated at Ondokuz Mayıs University Department of Oral and Maxillofacial Surgery between 2003 and 2010. Data were collected from patient files in the archive and were analyzed using SPSS version 20.0 software.

**Results:**

A total of 82 patients with 133 mandibular fractures were included in this study. After the follow up period of the patients, the results were achieved from 58 (70.7%) males and 24 (29.3%) females, whose ages ranged from 5 to 72 years and the mean age was 29. Fractures were most seen in 2008 and the busiest month was August. Falls (40.2%) were the major causes of mandibular fractures followed by traffic accidents and violence. The mandibular anatomical sites of higher fracture incidence were: condyle (34.6%), body and symphysis. The number of the fractures and injuries which were seen in other places such as zygomatic arch, alveolar process, tongue, upper and lower lips, orbita, arms was 14. 53 (64.6%) patients were treated by closed reduction, whereas 13 (15.8%) patients were treated by open reduction.

**Conclusions:**

We concluded that our results were widely similar with the studies in developing countries. Socio-economic factors, cultures, geographic conditions and education could affect the etiology of the mandibular fractures and cause different results between the studies conducted in different countries.

** Key words:**Mandibular fractures, etiology, trauma, treatment, complication.

## Introduction

Facial zone is the most fractured area in the body and mandible is one of the most frequent facial bones to be fractured because of the prominence, position and anatomic configuration. Among all of the maxillofacial fractures, mandible fracture rate was reported as 36% to 59% (1). The etiology of jaw fractures has been the topic of many studies. Violence is the most frequent etiologic factor in developed countries while a traffic accident is the major factor in countries. This situation is due to the differences in socioeconomic factors, geographic situations, religion, traffic rules and seasons among countries (2). 

This study was performed to analyze various aspects of mandibular fractures in Black Sea Region. Ondokuz Mayıs University Dental Faculty (OMUDF) has served to northern part of Anatolia alone for a long time and data of this study belong to the patients with mandibular fractures, who were referred to Ondokuz Mayıs University Dental Faculty between 2003 -2010.

## Material and Methods

In this study we retrospectively analysed 82 patients with 133 mandibular fractures in OMUDF between 2003-2010. Age, sex, fracture etiology, anatomic localization, monthly distribution of traumas and treatment methods were examined. Informed patient consent was obtained from the patients. Localization of the mandibular fracture were divided into seven groups such as symphysis, parasymphysis, body, angulus, ramus, condyle, coronoid and alveolar process fractures. Etiologic factors were evaluated under the titles of traffic accidents, falls, violence, sport accidents, oral pathologies, and iatrogenic factors. Data were collected from patient files in the archive and were analyzed using SPSS version 20.0 software (IBM, Armonk, NY, USA). Descriptive statistical methods (mean, standard deviation, and percent) were applied to data, and chi-square and Kruskal-Wallis tests were employed to assess mean differences using SPSS 20 statistical software.

## Results

-Age and Gender Distribution

In this retrospective study, 82 patients with 133 mandibular fractures were evaluated. It was found that the age range of patients was 5-80 and mean age were 29.1, STD 17.6 median 24. These values were 6-80 and 32.4 in women and 5-71 and 27.7 in men, respectively. There were 58 (70.7%) male and 24 (29.3%) female patients and M/F ratio was 2.41. There was no significant differences between sex and etiology (Chi-Square, *p*=0.215).

-Etiology

It was observed that the most common etiologic factors are falls 33 (40.2%), traffic accidents 20 (24.4%), violence 18 (22%), injuries with objects 5 (6.1%), iatrogenic factors 3 (3.7%), sport injuries 2 (2.4%) and pathologies 1 (1.2%) in our study ( Table 1). Relation between age and etiology was analyzed by Kruskal-Wallis but no significance was obtained (*p*=0.392).

**Table 1:**
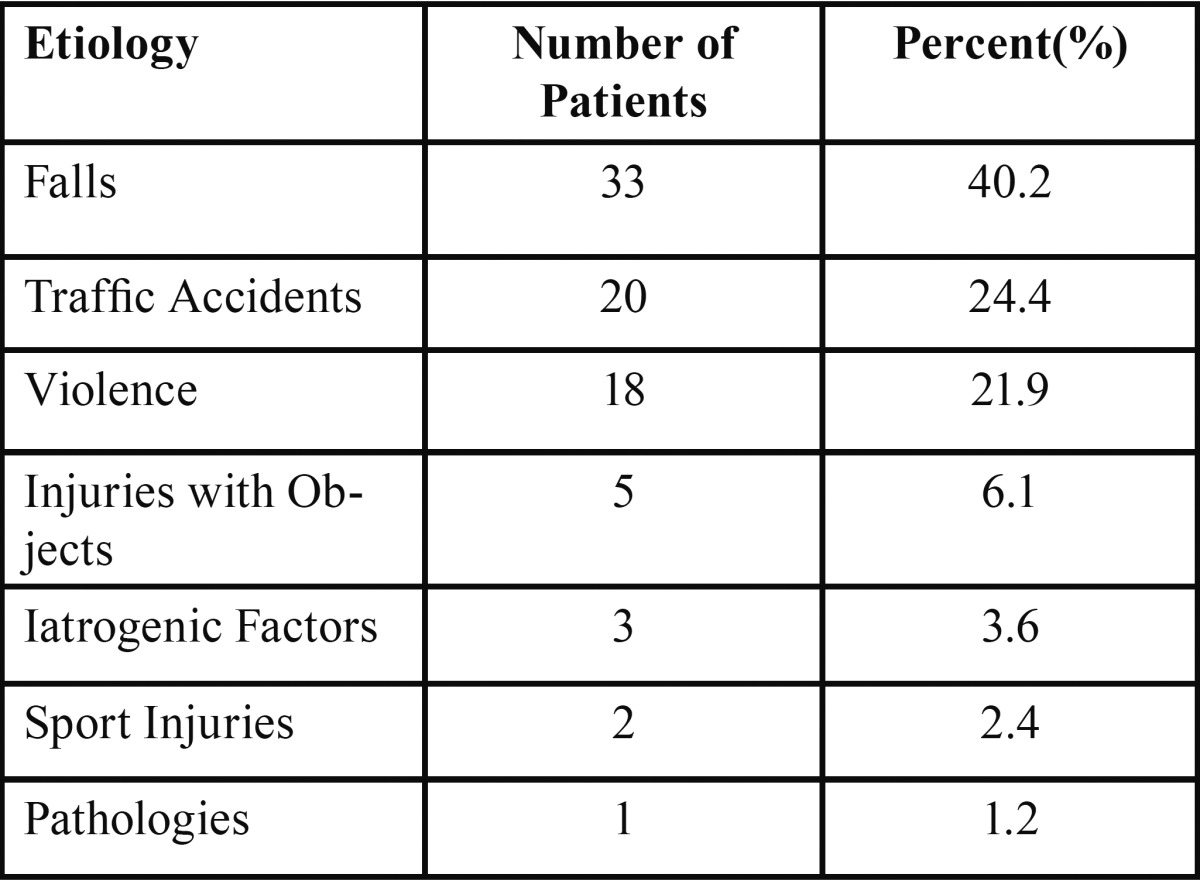
The distribution of the etiologic factors.

-Fracture Localization

**Figure 1:**
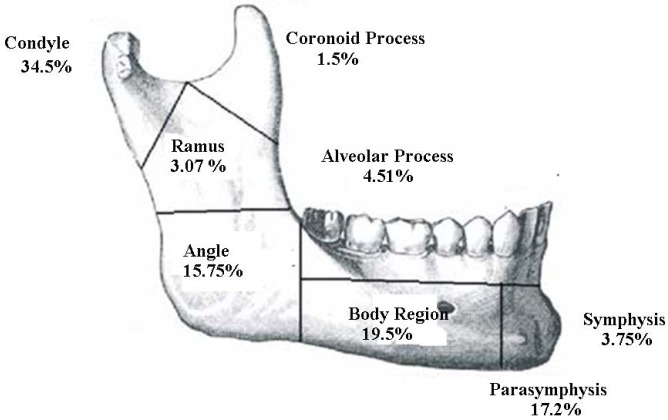
Anatomic localization of the fracture sites.

The most common site of fracture is condyle (34.5%) followed by body (19.5%) and parasymphysis (17.2%) regions. Configuration was shown in figure 1. No significance was obtained for the relationship between localization of mandible fracture and etiology (Chi-Square, *p*=0.708).

-Distribution by Months

Mandibular fractures were most seen in August (15.8%), which is followed by May (13.4%). In the winter months, number of fractures reduces (13.8%) ( Table 2). No signification was detected when etiology of fracture and its distribution by months were compared (Chi-Square, *p*=0.102).

**Table 2:**
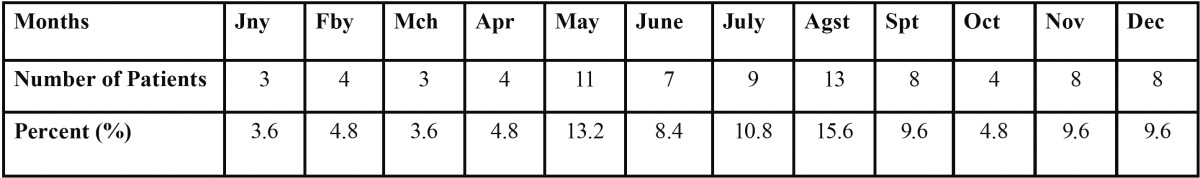
The distribution of the fractures according to seasons.

-Type and Number of the Fracture 

There were 11 (13.4%) patients with multiple fracture and 71 (86.6%) patients with isolated fracture. One fracture line in 41 (50%) patients, 2 fracture lines in 35 (42.6%) patients, 3 fracture lines in 5 (6.1%) patients and 4 fracture lines in 1 (%1.2) patient was observed. Relationship between fraction number and etiology was not statistically important.

-Management 

**Figure 2:**
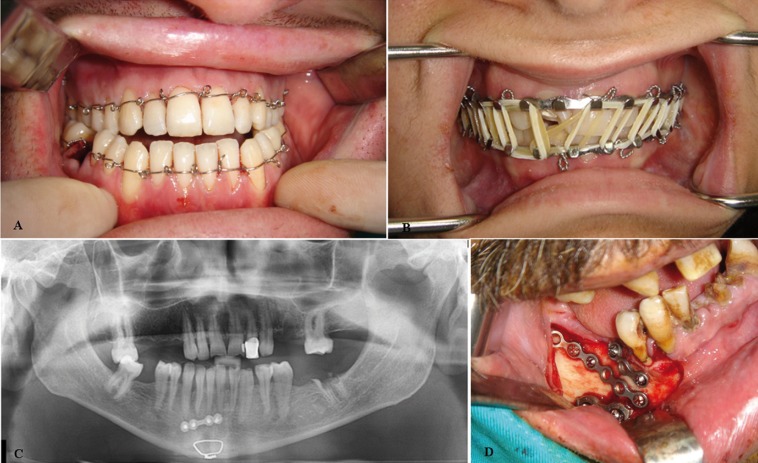
Different treatment protocols shown in pictures; A) IVY loop, B) Arch bar, C) Mini plate and wire osteosynthesis, D) Different mini plates applications.

Treatment protocol was shown in  Table 3. 44 (53.6%) patients were treated with closed reduction and 5 (6.1%) were treated with open reduction. Different techniques were used for patients who underwent closed and open reduction (Fig. 2). In closed reduction group, 39 of the patients were treated with arch bar, 2 of them with Ivy loop and 3 of them with splint. In open reduction group, 4 patients were treated mini plates and 1 wire osteosynthesis. Soft diet and medical treatment were administered to 15 (18.3%) patients in combination. 11 of them could not be treated because they were referred to our clinic too late. 7 patients were referred to other clinics because of the fracture in the critical areas of the body ( Table 3). 

**Table 3:**
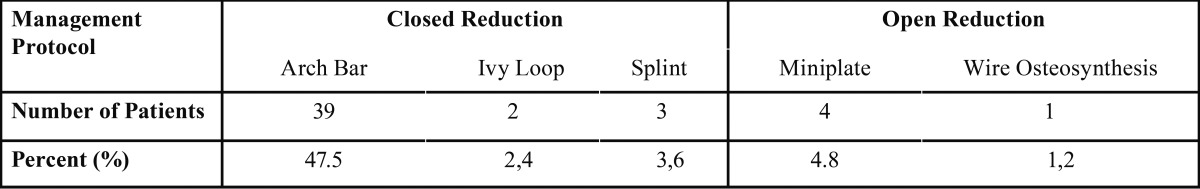
The distribution of the treatment protocols according to cases.

-Complications

Malocclusion in 12 patients and loss of sensation was observed in 3 patients.

## Discussion

Many authors have reported that mandible is the most frequent facial bone to be fractured (1-4). Most of the previous studies reported higher mandibular fracture incience in males (5-7). Borhman *et al.* (8) reported high incidence of mandibular fractures in young patients and the male: female ratio of 2.9:1. Schön *et al.* (9) reported a M:F ratio of 4:1. Elgehani *et al.* (10) reported M:F ratio of 7.1:1. Subhashraj *et al.* (11) reported a M:F ratio of 5.1:1.Matos *et al.* (12) reported a ratio of 3.7:1. In consistent with most of the studies in the literature, male:female ratio was found as 2.56 in our study (5-7).

Elgehani *et al.* (10) and Matos *et al.* (12) reported higher incidence of mandibular fracture in age group between 21 to 30 years. In contrast to these studies, Sakr *et al.* (2) reported higher mandibular fracture incidence between 0-10 years. Similarly, we also found higher mandibular fracture incidence in age group 11-30 (9,11,13). As mentioned before, because the children are generally under parental care, they are prevented from severe injuries and the elasticity of bones makes them less susceptible to fracture.

Many authors reported the angle as the most frequently affected site (14-16), whereas others reported this to be the mandible body (6,17), and symphysis (18). In contrast, Matos *et al.* (12) and Schön *et al.* (9) reported the condyle as the most frequently affected site. Ajmel *et al.* (19) reported that the parasymphyseal and body region were the most affected sites. Elgehani *et al.* (10) found that the most common site of fracture was the parasymphysis, followed by angle of the mandible. In the present study condyle is found to be the most common site (34.6%) followed by body (19.2%) and parasymphysis (18.4%) regions. The most common combination of bilateral or multiple fractures in our study is condyle with parasymphysis in 14 patients. This may be related to horizontally directed impact to the parasymphysis that led to the concentration of the tensile strain at the condylar neck resulting in condylar fracture.

In the literature, fights are reported to be the most common cause of mandibular fractures in rural and farming population compared to falls (17.2%) and motor vehicle accidents (10.9%) (5-7). Sakr *et al.* (2) reported that traffic accidents are the most common etiology for mandibular fractures in developing countries, whereas sport accidents are the most common cause in developed countries, where traffics laws are more widely respected. In this study, falls were the most common cause of mandibular fracture followed by traffic accidents and this result is similar with the developing countries. This may be because female patients reported fall as the most common reason for their injuries, indicating a high incidence of violence against girls and women. In this study, interestingly, 10 of 17 female patients reported fall as the reason for their injuries, the rest of the 17 female patients had a traffic accident.

In our study, distribution of the mandibular fractures according to the seasons and months were evaluated. We concluded that the busiest year was 2009 and the busiest month were August (15.8%) followed by May (13.4%). Sakr *et al.* (2) reported that the busiest month was January. We considered that the rising number of the mandibular fractures in the summer can be related with the visits of our expatriate people living in other countries. Also, outdoor activities have become more crowded in our country in the summer

Kirk *et al.* (13) treated 60.1 percent of the patients closed reduction (CR) and 30.9 percent open reduction (OR). Sakr *et al.* (2) treated 48 percent CR and 36.2 percent OR. Schon *et al.* (9) treated 105 patients ORIF and 9 patients CR. In the present study 5 patients treated OR, 44 CR. Soft diet and medical treatment were applied to 15 patients. Because of the late reference, 11 patients could not be treated. Seven patients were referred to other hospitals because of the fractures in the vital areas.

## Conclusions

We concluded that falls were the main cause of the mandibular fractures and this was followed by traffic accidents similar with other developing countries. We think this can be attributed to obtaining a wrong history from women with fractures. As mentioned before, especially domestic violence is reported as fall. It is presumed that many patients subjected to violence described the cause of fractures as a fall.

In the future we believe that the incidence of falls and traffic accidents will reduce according to the developed education and traffic rules.
